# Sports Radiology: A Key Driver of Clinical Decision-Making

**DOI:** 10.3390/diagnostics15151977

**Published:** 2025-08-07

**Authors:** Francesco Feletti

**Affiliations:** 1 Dipartimento di Medicina Traslazionale e per la Romagna, Università Degli Studi di Ferrara, 44121 Ferrara, Italy; francesco.feletti@unife.it; 2 EXTREMESPORTMED^®^, International Society of Medicine for Outdoor Physical Activity and Extreme Sports, 40026 Bologna, Italy

In recent years, radiology has become a key factor in sports clinical decision-making, primarily due to two parallel trends: the need for timely imaging in elite sports and the increase in recreational and weekend exercise-related injuries among individuals who adopt a healthy lifestyle, often in outdoor settings and without structured physical preparation.

At the professional level, during the Tokyo 2020 Summer Olympics (held in 2021), 567 MRIs, 352 radiographs, and 39 ultrasounds were performed at the Olympic Village Polyclinic [1], while approximately 300 MRIs were performed during the Paralympics [2]. Notably, although most imaging studies focused on the musculoskeletal system, they were also requested for suspected cases of appendicitis, cholecystitis, and hemosputum, demonstrating that modern elite sports radiology extends beyond the musculoskeletal system [3].

Similarly, during the 2022 FIFA World Cup in Qatar, a centralised medical facility performed 143 imaging exams on 94 athletes, most of which were MRI (67%), along with plain radiography, ultrasound, and CT. Acute muscle tears accounted for the majority of findings (42%), with imaging guiding both diagnosis and interventional procedures [4]. The medical services report by FIFA clinicians highlighted the critical role of centralised radiologic infrastructure integrated into multidisciplinary medical teams [5], thus confirming the transition of radiology from a supporting tool to a central component of clinical workflow in high-performance sport.

Nowadays, diagnostic imaging has become a cornerstone in the timely management of sports injuries in elite athletes, with two distinct purposes:

1. Facilitating an early return to play;
2. Reducing the risk of complications associated with delayed intervention.

Regarding the first purpose, a paradigmatic example is represented by the injury suffered by the Italian Olympic and World Cup alpine skiing champion Sofia Goggia, in December 2022 during a World Cup downhill race in St. Moritz.

An impact with a gate pole resulted in fractures of the left second and third metacarpal bones. Radiological assessment performed at a local clinic enabled same-day surgical management with internal fixation using plates and screws, allowing the athlete to return to competition the very next day. Remarkably, she went on to win the downhill event less than 24 h after the injury [6].

As for the second purpose, the NBA professional basketball player Jayson Tatum (of the Boston Celtics) experienced a right Achilles tendon injury during the NBA Eastern Conference Semifinals between the Boston Celtics and the New York Knicks at Madison Square Garden in May 2025.

The injury was caused by a sudden deceleration following a penetration to the basket. An MRI examination performed on the same day demonstrated a full-thickness tear. The accurate and quick MRI diagnosis provided critical information for clinical decision-making. It allowed for surgical repair within 24 h, optimising postoperative outcomes and minimising the risks associated with delayed treatment [7,8,9].

Beyond elite participants in traditional sports, outdoor physical activity and extreme sports—typically practised by weekend warriors in aerial, water, mountain, and urban environments—account for 3% of major trauma cases (defined as an Injury Severity Score of 12 or greater) in three western Canadian regions [10].

These injuries frequently require emergency radiology, as they may involve a wide range of anatomical injury distributions affecting the head (55.8%), spine (35.0%), thorax (35.0%), extremities (31.1%), face (17.4%), and abdomen (13.1%) [10].

Both first- and second-level imaging techniques, such as X-ray, sonography, computed tomography, and MRI, have been used to assess injuries associated with extreme sports [11]. These sports encompass a wide variety of types and often present with characteristic musculoskeletal and orthopaedic patterns [12].

Variations in imaging protocols can significantly affect clinical outcomes for athletes in different sports. The nature of injuries can differ widely— for example motor sports and contact sports may lead to more concussions [13,14] and some extreme sports may lead to high energy trauma in the case of mismanaged execution [11], necessitating different imaging techniques compared to sports that involve repetitive motion, such as swimming or running, where overuse soft tissue injuries may be more prevalent [15,16].

To improve diagnosis and treatment efficacy, it may be crucial tailoring imaging protocols to the specific demands and injury patterns of each sport. However, it requires in-depth knowledge of the typical injury dynamics in each sport.

The evolving landscape of sports practice—ranging from elite competition to the growing phenomenon of recreational and extreme outdoor physical activity—demands a radiological approach that is both responsive and forward-looking.

Diagnostic imaging is no longer a passive support tool but a pivotal element in clinical decision-making, potentially influencing management decisions, return to training and competition, and prediction of injury recurrence [17].

Recent advances in quantitative MRI, US, and CT offer objective insights into the pathophysiologic mechanisms of muscle, tendon, and ligament injuries in athletes. They could guide sports physicians in patient management by eventually supporting the prediction of return to play and the estimation of reinjury risk [18].

For example, contrast-enhanced US [19], as well as compositional and functional MRI techniques—including T2 mapping [20], diffusion tensor imaging [21,22,23], and sodium imaging [24]—have been applied to quantify pathophysiologic processes and biochemical compositions of muscles, tendons, ligaments, and cartilage.

Some studies suggest that shear-wave elastography [25] and MR elastography [26] may offer a non-invasive means to assess the mechanical, elastic, and physical properties of tissues involved in muscle and tendon injuries in sport.

Finally, dual-energy CT has been used to characterise injuries in high-speed winter sports, such as skiing, snowboarding, and skating, offering additional advantages over conventional CT, including the creation of quantitative bone marrow oedema maps, reduction in metal artefacts, and enhanced assessment of ligamentous injuries [27].

However, the future role of such novel imaging techniques remains to be established [14]. A major issue preventing the translation of these imaging tools to clinical practice is the lack of standardisation, including image acquisition and analysis, the absence of established normal values, and variability among scanners and imaging sites [28].

For these reasons, there is a need for prospective longitudinal studies with larger sample sizes to validate the added value of the aforementioned quantitative imaging methods [14].

Some technological advancements in sports radiology are currently being explored to enhance diagnostic accuracy. They include the adoption of higher-field-strength MRI scanners [29] and the development of artificial intelligence algorithms to provide more precise and more detailed images, as well as aid in image interpretation [30,31].

Additionally, the integration of real-time MRI enables the tracking of bone motion in real time, allowing for the acquisition of 3D reconstructions of joints during motion, consequently providing accurate quantitative biomechanical data [32] that further enhances the decision-making process for medical teams.

In conclusion, the changing healthcare needs associated with physical activity are making radiology increasingly essential to clinical management in sports medicine, enabling timely diagnosis, treatment planning, and recovery monitoring in both professional and amateur athletes. To be efficient, radiology services must ensure rapid access to tests, high diagnostic reliability, and integration into multidisciplinary care pathways. For the same reasons, integrating sports radiology into radiology training programmes is essential. A key aspect of this education should be the study of injury epidemiology and sport-specific trauma patterns, as well as the understanding of the biomechanical dynamics unique to each discipline. This knowledge enables radiologists to tailor imaging studies in a more targeted way—for example, by selecting specific projections or techniques based on the expected type of injury.

## Data Availability

Not applicable.

## Abbreviations

The following abbreviations are used in this manuscript:

MRI	Magnetic Resonance Imaging
CT	Computed Tomography
US	Ultrasound

## References

[1] Radiological Society of North America (RSNA) (2024). Radiology at the Olympics. RSNA News.

[2] Ward P. (2021). First Figures Released on Imaging of Tokyo Olympics. AuntMinnie.

[3] AuntMinnieEurope (2021). What Was the Big Surprise for Imaging Boss at Tokyo Olympics?. AuntMinnieEurope.

[4] Bordalo M., Serner A., Yamashiro E., Al-Musa E., Djadoun M.A., Al-Khelaifi K., Schumacher Y.O., Al-Kuwari A.J., Massey A., D’hOoghe P. (2023). Imaging detected sports injuries and imaging guided interventions in athletes during the 2022 FIFA football (soccer) World Cup. Skelet. Radiol..

[5] Schumacher Y.O., Kings D., Whiteley R., Dharman A., Taqtaq G., Mc Court P., Alkhelaifi K., Targett S., Holtzhausen L., E Pieles G. (2023). Medical services at the FIFA World Cup Qatar 2022. Br. J. Sports Med..

[6] de Villiers O. (2022). Sofia Goggia Wins St Moritz Downhill a Day After Hand Operation. Olympics.com.

[7] Rohrbach B. (2025). Jayson Tatum’s Achilles Injury Is a Devastating Blow to the Celtics’ Future—And a Star in His Prime. Yahoo Sports.

[8] Hartwell D. (2025). Tatum has Ruptured Achilles in Worst-Case Scenario for Celtics Star. NBC Sports Boston.

[9] Fonseca C. (2025). A Really Significant Injury: Jayson Tatum Ruptured His Achilles’ Tendon. Local Doctors Weigh in on What’s Next. The Boston Globe.

[10] Roberts D., Ouellet J.-F., McBeth P., Kirkpatrick A., Dixon E., Ball C. (2014). The “weekend warrior”: Fact or fiction for major trauma?. Can. J. Surg..

[11] Feletti F., Brymer E. (2018). Injury in kite buggying: The role of the ‘out-of-buggy experience’. J. Orthop. Surg. Res..

[12] Feletti F., Mei-Dan O., Canata G.L., Jones H. (2022). Extreme Sports. Epidemiology of Injuries in Sports.

[13] Feletti F., Brymer E. (2018). Pediatric and adolescent injury in skateboarding. Res. Sports Med..

[14] Bytomski J. (2022). Sports Medicine: Concussion. FP Essent.

[15] Boltz A.J., Robison H.J., Morris S.N., D’Alonzo B.A., Collins C.L., Chandran A. (2021). Epidemiology of Injuries in National Collegiate Athletic Association Men’s Swimming and Diving: 2014–2015 Through 2018–2019. J. Athl. Train..

[16] Arnold M.J., Moody A.L. (2018). Common Running Injuries: Evaluation and Management. Am. Fam. Physician.

[17] Guermazi A., Roemer F.W., Robinson P., Tol J.L., Regatte R.R., Crema M.D. (2017). Imaging of Muscle Injuries in Sports Medicine: Sports Imaging Series. Radiology.

[18] Hayashi D., Roemer F.W., Tol J.L., Heiss R., Crema M.D., Jarraya M., Rossi I., Luna A., Guermazi A. (2023). Emerging Quantitative Imaging Techniques in Sports Medicine. Radiology.

[19] Hotfiel T., Heiss R., Swoboda B., Kellermann M., Gelse K., Grim C., Strobel D., Wildner D. (2018). Contrast-enhanced ultrasound as a new investigative tool in diagnostic imaging of muscle injuries—A pilot study evaluating conventional ultrasound, CEUS, and findings in MRI. Clin. J. Sport Med..

[20] Zaeske C., Brueggemann G.P., Willwacher S., Maehlich D., Maintz D., Bratke G. (2022). The behaviour of T2 and T2 relaxation time in extrinsic foot muscles under continuous exercise: A prospective analysis during extended running. PLoS ONE.

[21] Hooijmans M.T., Monte J.R., Froeling M., Berg-Faay S.v.D., Aengevaeren V.L., Hemke R., Smithuis F.F., Eijsvogels T.M., Bakermans A.J., Maas M. (2020). Quantitative MRI reveals microstructural changes in the upper leg muscles after running a marathon. J. Magn. Reson. Imaging.

[22] Froeling M., Oudeman J., Strijkers G.J., Maas M., Drost M.R., Nicolay K., Nederveen A.J. (2015). Muscle changes detected with diffusion-tensor imaging after long-distance running. Radiology.

[23] Giraudo C., Motyka S., Weber M., Karner M., Resinger C., Feiweier T., Trattnig S., Bogner W. (2018). Normalised STEAM-based diffusion tensor imaging provides a robust assessment of muscle tears in football players: Preliminary results of a new approach to evaluate muscle injuries. Eur. Radiol..

[24] Dahlmann A., Kopp C., Linz P., Cavallaro A., Seuss H., Eckardt K.-U., Luft F.C., Titze J., Uder M., Hammon M. (2016). Quantitative assessment of muscle injury by (23)Na magnetic resonance imaging. Springerplus.

[25] Takenaga T., Sugimoto K., Goto H., Nozaki M., Fukuyoshi M., Tsuchiya A., Murase A., Ono T., Otsuka T. (2015). Posterior shoulder capsules are thicker and stiffer in the throwing shoulders of healthy college baseball players: A quantitative assessment using shear-wave ultrasound elastography. Am. J. Sports Med..

[26] Cloutier G., Destrempes F., Yu F., Tang A. (2021). Quantitative ultrasound imaging of soft biological tissues: A primer for radiologists and medical physicists. Insights Imaging.

[27] Hickle J., Walstra F., Duggan P., Ouellette H., Munk P., Mallinson P. (2020). Dual-energy CT characterisation of winter sports injuries. Br. J. Radiol..

[28] Chalian M., Li X., Guermazi A., Obuchowski N.A., Carrino J.A., Oei E.H., Link T.M., RSNA QIBA MSK Biomarker Committee (2021). SNA QIBA MSK Biomarker Committee Members. The QIBA profile for MRI-based compositional imaging of knee cartilage. Radiology.

[29] Menon R.G., Chang G., Regatte R.R. (2021). Musculoskeletal MR Imaging Applications at Ultra-High (7T) Field Strength. Magn. Reson. Imaging Clin. N. Am..

[30] Guermazi A., Omoumi P., Tordjman M., Fritz J., Kijowski R., Regnard N.E., Carrino J., Kahn C.E., Knoll F., Rueckert D. (2024). How AI May Transform Musculoskeletal Imaging. Radiology.

[31] Chen W., Lim L.J.R., Lim R.Q.R., Yi Z., Huang J., He J., Yang G., Liu B. (2024). Artificial intelligence powered advancements in upper extremity joint MRI: A review. Heliyon.

[32] Garetier M., Borotikar B., Makki K., Brochard S., Rousseau F., Ben Salem D. (2020). Dynamic MRI for articulating joint evaluation on 1.5 T and 3.0 T scanners: Setup, protocols, and real-time sequences. Insights Imaging.

